# Supplementary material for: The Effect of Dietary Carbohydrate and Fat Manipulation on the Metabolome and Markers of Glucose and Insulin Metabolism: A Randomised Parallel Trial
Source: Nutrients. 2022 Sep 7;14(18):3691. doi: 10.3390/nu14183691 (PMC9505524; doi:10.3390/nu14183691)
Supplement: Supplementary file 1 [file nutrients-14-03691-s001.zip › nutrients-1853323-supplementary.pdf]

Supplement S1 – Consort Checklist

CONSORT 2010 checklist of information to include when reporting a randomised trial*

Section/Topic	Item No	Checklist item	Reported on page No
**Title and abstract**			
	1a	Identification as a randomised trial in the title	1
	1b	Structured summary of trial design, methods, results, and conclusions (for specific guidance see CONSORT for abstracts)	1
**Introduction**			
Background and objectives	2a	Scientific background and explanation of rationale	2
	2b	Specific objectives or hypotheses	2
**Methods**			
Trial design	3a	Description of trial design (such as parallel, factorial) including allocation ratio	2-3
	3b	Important changes to methods after trial commencement (such as eligibility criteria), with reasons	2-3
Participants	4a	Eligibility criteria for participants	2-3
	4b	Settings and locations where the data were collected	3
Interventions	5	The interventions for each group with sufficient details to allow replication, including how and when they were actually administered	2-3
Outcomes	6a	Completely defined pre-specified primary and secondary outcome measures, including how and when they were assessed	3-4
	6b	Any changes to trial outcomes after the trial commenced, with reasons	n/a
Sample size	7a	How sample size was determined	n/a
	7b	When applicable, explanation of any interim analyses and stopping guidelines	n/a
Randomisation:			
Sequence generation	8a	Method used to generate the random allocation sequence	3
	8b	Type of randomisation; details of any restriction (such as blocking and block size)	3
Allocation concealment mechanism	9	Mechanism used to implement the random allocation sequence (such as sequentially numbered containers), describing any steps taken to conceal the sequence until interventions were assigned	3
Implementation	10	Who generated the random allocation sequence, who enrolled participants, and who assigned participants to interventions	3
Blinding	11a	If done, who was blinded after assignment to interventions (for example, participants, care providers, those assessing outcomes) and how	n/a
	11b	If relevant, description of the similarity of interventions	n/a

Statistical methods	12a	Statistical methods used to compare groups for primary and secondary outcomes	4-5
	12b	Methods for additional analyses, such as subgroup analyses and adjusted analyses	4-5
**Results**			
Participant flow (a diagram is strongly recommended)	13a	For each group, the numbers of participants who were randomly assigned, received intended treatment, and were analysed for the primary outcome	5
	13b	For each group, losses and exclusions after randomisation, together with reasons	5
Recruitment	14a	Dates defining the periods of recruitment and follow-up	n/a
	14b	Why the trial ended or was stopped	n/a
Baseline data	15	A table showing baseline demographic and clinical characteristics for each group	6
Numbers analysed	16	For each group, number of participants (denominator) included in each analysis and whether the analysis was by original assigned groups	5-9
Outcomes and estimation	17a	For each primary and secondary outcome, results for each group, and the estimated effect size and its precision (such as 95% confidence interval)	5-9
	17b	For binary outcomes, presentation of both absolute and relative effect sizes is recommended	5-9
Ancillary analyses	18	Results of any other analyses performed, including subgroup analyses and adjusted analyses, distinguishing pre-specified from exploratory	5-9
Harms	19	All important harms or unintended effects in each group (for specific guidance see CONSORT for harms)	9
**Discussion**			
Limitations	20	Trial limitations, addressing sources of potential bias, imprecision, and, if relevant, multiplicity of analyses	11
Generalisability	21	Generalisability (external validity, applicability) of the trial findings	9-11
Interpretation	22	Interpretation consistent with results, balancing benefits and harms, and considering other relevant evidence	9-11
**Other information**			
Registration	23	Registration number and name of trial registry	3
Protocol	24	Where the full trial protocol can be accessed, if available	n/a
Funding	25	Sources of funding and other support (such as supply of drugs), role of funders	12

*We strongly recommend reading this statement in conjunction with the CONSORT 2010 Explanation and Elaboration for important clarifications on all the items. If relevant, we also recommend reading CONSORT extensions for cluster randomised trials, non-inferiority and equivalence trials, non-pharmacological treatments, herbal interventions, and pragmatic trials. Additional extensions are forthcoming: for those and for up to date references relevant to this checklist, see www.consort-statement.org.

Table S1: Cardiometabolic risk scoring system.

**Cardiometabolic measure**	**Points**
Fasting glucose: > 5.5 mmol/L	3
Fasting triglycerides: > 1.3 mmol/L	1
Fasting HDL-C: < 1.0 mmol/L (males), < 1.3 mmol/L (females)	2
Waist circumference: > 94 cm (males), > 80 cm (females)	1
Waist circumference: > 102 cm (males), > 88 cm (females)	2
Systolic blood pressure: > 130 mmHg	1
Diastolic blood pressure: > 85 mmHg	1

Supplement S2 – LCHF dietary guide

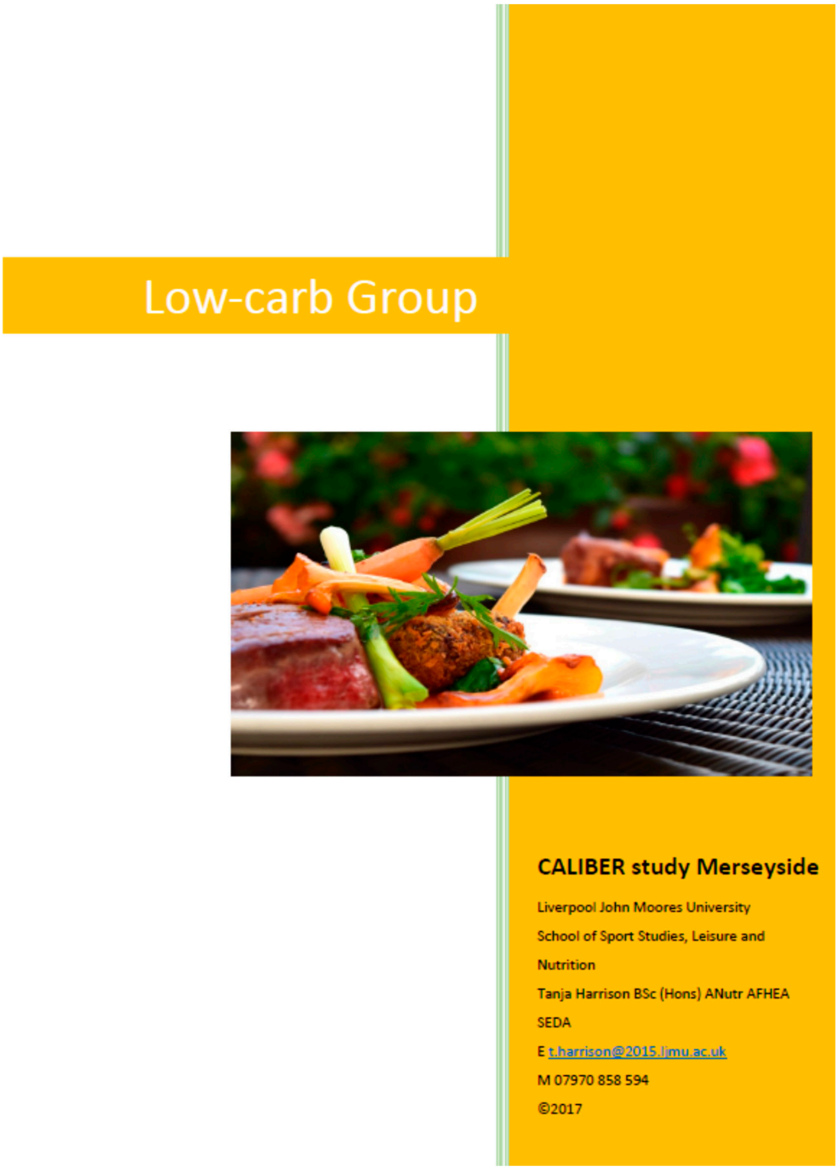
Low-carb Group

CALIBER study Merseyside

Liverpool John Moores University

School of Sport Studies, Leisure and Nutrition

Tanja Harrison BSc (Hons) ANutr AFHEA SEDA

E t.harrison@2015.ljmu.ac.uk

M 07970 858 594

©2017

Contents

Quick guide 3
Introduction 4
What do we want to find out? 4
What to expect during you visits to our lab 5
Your involvement in the study – Flow chart low-carb group 7
Your appointment schedule 8
Your contacts 9
Further information throughout the study 9
Following a low-carb diet – What does that actually mean? 10
Potential beneficial effects of a low-carb diet 10
Potential adverse effects of a low-carb diet 11
How to take your multivitamin supplement 11
Portion sizes for carbohydrates 12
What is fibre and why are we supposed to eat it? 13
Carbohydrate and fibre content of fruit and vegetables permitted on a low-carb diet 14
Carbohydrate and fibre content of nuts and seeds 16
Permitted foods on a low-carb diet 16
Dietary fat – the star of the show? Following a high-fat diet 16
Proteins to help you build and maintain that temple which is your body 17
Animal-based proteins 18
A word on dairy products 19
Plant-based proteins 19
Portion sizes for protein 20
Vegetables 20
Fruit 20
Magnesium-rich foods 21
Potassium-rich foods 21
Food no-no's on a low-carb diet 21
So what should you eat in a day if you are following a low-carb, high-fat diet 22
Suitable snacks 23
Some staples and alternatives to favourites on a very low-carb diet 23
Hydration 24

Alcohol 24
Learning to read labels and nutritional information 24
Label reading 24
Labels at the back of the packaging 25
Front of pack labels 27
Interpreting nutritional information provided by fast food restaurant and pub chains 27
Tips for Eating Out on a low-carb, high-fat diet 27
Appendix - Helpful App to help you to stick to a low-carb diet 29
Change4Life – Sugar smart App 29
Appendix - Examples nutritional information fast food, pub and restaurant chains 30
ASK Italian 30
Brewers Fayre 31
Five Guys 32
Harvester 33

Introduction

Hello and welcome to the CALIBER study! Nice to have you on board.

This booklet has been designed to be your companion over the next eight weeks whilst you are part of our cohort and to support you to consume a healthy diet whether you are cooking at home, buying ready-made meals or eating out. There are certain rules that can be applied to all of these situations.

We will also give you information on the purpose of our study, what to expect during your visits to our laboratories and once the study has finished and we have analysed the results. This means that not only will you help an important research cause but you can also find out how you and your body did over the course of these eight weeks and how your body composition, blood profile, food cravings and cognition might have been affected.

We hope that you will find your time on the study interesting, inspiring, motivating and delicious.

What do we want to find out?

Maybe you have followed the news over these past couple of years and noticed that there is a lot of controversy and discussion about what makes a diet healthy. The debate has been particularly heated around the issue of carbohydrates. Whilst many public health officials have argued that the vast majority of the population does not follow the dietary UK guidelines (which can be classed as high-carbohydrate, moderate-fat) and that this is the root cause of the UK's problem with ill-health, obesity and diseases such as heart disease and type 2 diabetes, others claim that these guidelines have caused these problems to begin with. The latter group advocates to reduce the amounts of carbohydrates we consume as a nation and for the guidelines to be re-written.

A third group suggests that it is far more complicated than this and that how we react to carbohydrates is actually far more personal and not one-size-fits-all, but some people might be better off on low-carb diets whilst others fare better on high-carb diets in terms of cardiometabolic health.

This is where our study comes in, CALIBER – Carbohydrates, lipids and biomarkers of traditional and emerging cardiometabolic risk factors. We want to compare the effects of consuming either a high-carb or a low-carb diet on these risk factors. The reason why we have asked you to join us is because you showed some slightly elevated risk markers for these illnesses, albeit still at a stage where these should be easy to control and improve through a healthy diet, be this in form of low-carb, high-fat or high-carb, moderate-fat.

The discussion surrounding carbohydrates does not simply stop at their potential impact on risk markers that we find in our blood. There is also some discussion whether carbohydrates or fats lead to an increase in waist circumference and the development of fat deposits in our bodies, whether carbohydrates or fats are the root cause of food cravings that we might experience and whether one or the other somehow helps us to move about more or makes us more sluggish.

As scientists and nutritionists we are naturally curious to find out what might be going on. So thank you for joining us!

What to expect during you visits to our lab

There will be three appointments where we will ask you to come to IM Marsh campus (L17 6BD) for more thorough assessments, one right at the start when you will commence to eat according to the guidelines given to you, one after about 4 weeks and the final visit 8 weeks after your first one.

Each visit is expected to last between 60 and 90 minutes and will entail

A venous blood sample

We take about 8 teaspoons of blood. – Please note that you will have to have fasted for at least 12 hours prior to your appointment as otherwise your blood sugar and your triglycerides might be far higher than normal – painting a wrong picture of how the eating plan is working for you. Just as you had to do before coming in for your initial finger prick appointment you will also have to restrain from drinking alcohol or undertaking any strenuous exercise the night before. Again both can have an impact on your blood profile! We will analyse this blood sample at the end of the study to see how any risk factors for heart disease and type 2 diabetes might have changed over the course of eight weeks.

Assessing your body composition

This will be done in two different ways. Firstly, we will use a tape measure to measure your waist circumference, hip circumference, thigh circumference, calf circumference and neck circumference as these are all sites on the human body that can give us clues about the overall distribution of body fat. – **Please ensure that you bring a pair of shorts with you to these** visits as we will ask you to change into these before we take these measurements. If you prefer for a team member of the same sex to take these, please do let us know so that we can ensure that this can be facilitated.

Secondly, we will ask you to step onto sophisticated body composition scales (far bigger than the common bathroom ones) and measure your lean body mass, your body fat mass and the amount of fat surrounding your organs.

Taking your blood pressure

As blood pressure has been found to be an important factor in cardiometabolic health, we will assess your blood pressure every time you come to see us in our labs. Following standard protocol, we will take your blood pressure three times at each appointment and calculate the average of these three.

Going through a couple of brief questionnaires with you and conducting one final interview

During your second and your final visit we will go through a check list to see which types of fibre-containing foods you have consumed over the previous four weeks. We will also conduct a brief interview to check whether you have experienced any so-called adverse events over the past 4 weeks whilst you were eating according to the low-carb rules.

We will also ask you to bring the container containing your multivitamin and mineral supplement with you containing any remaining pills. During your second lab visit we will provide you will a further supply of supplements for the final four weeks of the study.

During your final visit we will also ask to stay with us for a little longer to conduct a brief interview with you asking you about your experiences with the diet allocated to you.

Prior to your lab appointments – recording of physical activity

On three occasions (just before your first, second and final visit to our lab) we will ask you to wear a physical activity monitor that looks like a digital watch and has to be worn on the wrist (just like a 'Fit bit') of your non-dominant arm. That means if you are right-handed you will have to wear this on your left wrist.

We will give you this device at least 8 days before your visits to our labs and will ask you to start wearing it for 7 days and at least 10 hours per day commencing on the morning after it has been handed to you and finishing the night before your lab appointment. During this time you will have to complete a wear-time diary on a daily basis, in which you will briefly record the times you are putting the monitor on in the morning, the times you are taking it off at night and any time during the time when you need to remove and put it back on, for example when you are taking a shower or when you are going swimming.

You will need to bring the monitor and the wear time diary with you on the morning of your lab appointment where the research team can collect it from you.

Prior to your lab appointments – food diaries and questionnaires

When you are given your physical activity devices we will also give you a template of a four-day food diary, which you will need to complete for four days before your lab appointment and bring with you on the morning.

We will also give you a number of brief questionnaires, which will assess your cognition and your food cravings over the previous four-week period. These will not take long to complete. Please also bring these questionnaires with you to your lab appointment and pass them on to our research team.

Your involvement in the study – Flow chart low-carb group

8 days prior to first lab visit

- Get physical activity monitor and wear for 7 days
- Complete 4-day food diary
- Complete food cravings questionnaire and cognition questionnaire

First lab visit

- Provide blood sample
- Undergo body composition assessment
- Take blood pressure

8 days prior to second lab visit

- Get physical activity monitor and wear for 7 days
- Complete 4-day food diary
- Complete food cravings questionnaire and cognition questionnaire

Second lab visit

- Provide blood sample
- Take blood pressure
- Undergo body composition assessment
- Complete fiber-intake interview, adverse events interview and multivitamin count

8 days prior to final lab visit

- Get physical activity monitor and wear for 7 days
- Complete 4-day food diary
- Complete food cravings questionnaire and cognition questionnaire

Final lab visit

- Provide blood sample
- Take blood pressure
- Undergo body composition assessment
- Complete fiber-intake interview, adverse events interview and multivitamin count
- Complete interview on experiences with low-carb, high-fat diet

Post-study

- Personalised dietary consultation based on your blood test results, body composition assessments and completed questionnaires

Physical activity monitor collection//delivery	First lab visit	Physical activity monitor collection/ /delivery	Second lab visit	Physical activity monitor collection/ /delivery	Third lab visit

All lab visits will take place at LJMU IM Marsh campus in Mossley Hill (L17 6BD).

Your contacts

Your main contact is Tanja Harrison who is also a registered associate nutritionist with the Association for Nutrition.

M 07970 858 594

E T.harrison@2015.ljmu.ac.uk

If you have any queries or concerns throughout the study, please do not hesitate to contact Tanja.

Other researchers on the team that you will meet on a regular basis

Deaglan McCullough

E d.mcullough@2015.ljmu.ac.uk

Dr Ian Davies (A registered nutritionist, focus on nutrition science)

E i.g.davies@ljmu.ac.uk

Dr Katie Lane (A registered nutritionist, focus on food)

E k.e.lane@ljmu.ac.uk

Dr Kevin Enright

E k.j.enright@ljmu.ac.uk

This study was approved by LJMU's Research Ethics Committee on 16 December 2016 (Ref. 16/ELS/029). If you any concerns regarding your involvement in this research, please discuss these with the researcher in the first instance. If you wish to make a complaint, please contact researchethics@ljmu.ac.uk and your communication will be re-directed to an independent person as appropriate.

Further information throughout the study

You can also find all the materials in a private Facebook Group, which you can request to join should you wish to do so at https://www.facebook.com/groups/lowcarbCALIBER/

We will also post regular updates here. However, should you not wish to join all the links and information will be provided to you via email or in hard copy.

Following a low-carb diet – What does that actually mean?

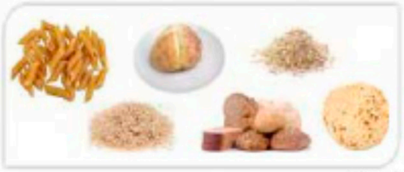

This guideline has been designed to help you follow a low-carbohydrate, high-fat diet over the next eight weeks whilst you are part of our cohort.

The diet that has been assigned to you means that you will eat only small amounts of carbohydrates per day and a higher proportion of fat. Whilst you are taking part in study we ask you to consume a minimum of 30 grams and a maximum of 50 grams of carbohydrates per day.

On a usually consumed Western diet the human body uses the glucose derived from carbohydrates for nearly all its energy needs. However, when carbohydrate intake is restricted the body learns to adapt very quickly and uses dietary and body fat instead. This happens through a process called "ketosis", those fatty acids consumed with our foods and those stored within our body fat cells are broken down in order to produce glycerol and ketone bodies. On very low-carb diets these products will provide the energy required by the human body for normal functioning.

In terms of your diet this means that you will instead eat more fat which will come from a variety of sources.

Carbohydrates come from a range of foods, including potatoes, rice, pasta, bread, fruit and vegetables and to a lesser extend dairy products (in the form of milk sugar, called lactose). This means that there will be restrictions as to which and how much of these foods you will be able to consume.

Potential beneficial effects of a low-carb diet

There is a reason why a growing number of nutrition and health professionals are in favour of a low-carb, high-fat diet.

- You should feel less hungry, especially between meals
- This means that you might eat less than you usually do, leading to a lower calorie intake and consequential potential weight loss
- Your body composition might change meaning less fat mass
- Your insulin levels should decrease as your body needs to produce less insulin to maintain blood sugar levels after a meal
- You will have better blood sugar control, which means that your body needs to produce less insulin to ferry the glucose in your blood into your muscles and you fat depots
- Your cravings for sugary and fatty foods should reduce which means that you will probably eat less of these foods.
- You might feel less bloated

Potential adverse effects of a low-carb diet

However, as we have already told you when you signed up – remember that disclaimer? – some people might have a bit of a harder time adjusting to the new diet. Especially during the first few days on a very low-carb diet you might experience

- Headaches - Drink plenty of fluids.
- Lethargy, weakness - If this is the case try and take it easy for a few days.
- Constipation - This is due to the potentially lower fibre intake. You can address this by increasing your fibre intake and drinking plenty of fluids. Please see the list of fibre-rich permitted foods on page 15.
- Muscle cramps -This is due to your kidneys excreting more sodium which can also impact on your potassium balance. Sodium and potassium are minerals which are used by your body as electrolytes, meaning they regulate the fluid balance in our bodies and stimulate our muscles and nerves. You can deal with this by continuing to take your daily multivitamin and mineral supplement provided by us and by drinking cups of bouillon or home-made bone broth (see recipe). You can also add half a teaspoon of salt to one litre of water and drink this throughout the day. In addition, consume plenty of magnesium and potassium-rich foods from the list provided.
- Brain fog – As your brain has used carbs so far to do all its hard work the initial switch from one fuel (carbs) to another (fats) might take a few days. If you can, try and take it easy for a few days and drink plenty of fluids.
- Nausea, anxiety and palpitations - This might also be due to your electrolyte deficiency (Magnesium and potassium).
- Poor sleep quality – After a while, however, a lot of people report a better quality of their sleep
- Bad breath – A sugar-free mint might help. Bad breath is a sign of your body switching from using carbs to fats (ketones) as fuel source. It will pass!
- Consumption of potentially fewer vitamins and minerals – This is why we are giving you your daily multi-vitamin and mineral supplement.

For these reasons we are actually advising you to start the diet on a less busy day of your week.

How to take your multivitamin supplement

During your first lab visit when you give your first venous blood sample we will also provide you with a multivitamin and mineral supplement, which you will need to take on a daily basis. The container includes 30 pills, which should be sufficient until your second lab appointment where you will receive the supplements for the following 4 weeks.

Please bring the pill container with you to each lab appointment, as we will need to count the number of pills left in the container. We are doing this as the number of pills taken will be taken into account when we analyse the data. It will also help us to explain why some side effects might have occurred – for example if you forgot very often to take your supplement.

Please do remember to take it on a daily basis. Preferably at mealtimes as some of the vitamins can only be absorbed by our bodies when consumed at the same time as fat. Having said this, the other vitamins will also be more readily available to your body when other nutrients have to be digested with them.

Portion sizes for carbohydrates

So, what do 50 grams of carbs look like?

As there are foods that are naturally high in carbs and those that are naturally low in carbs it can be confusing to decide how much you could eat of these foods.

Below you can see examples of what 50 grams of carbs might look like and how easy it would be to exceed your daily allowance.

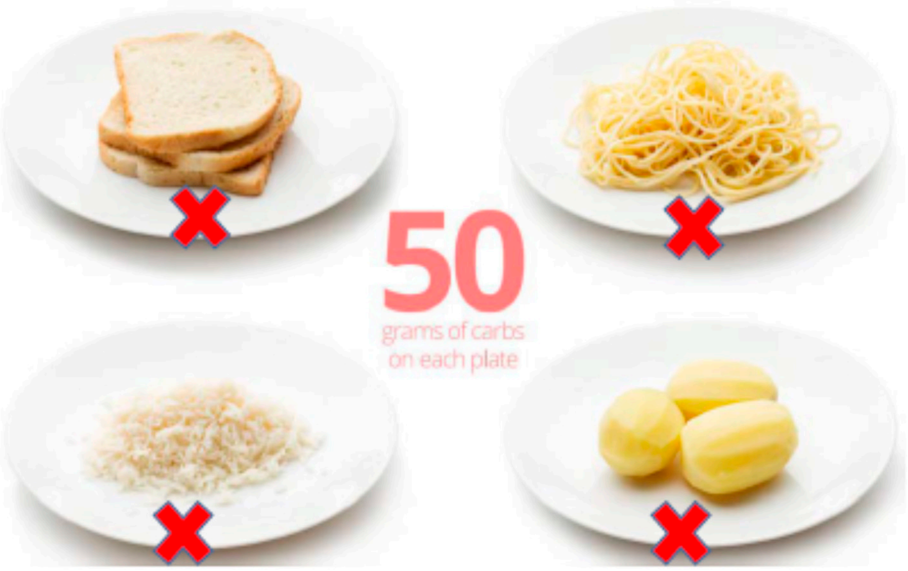

(Source: The Dietdoctor, 2017)

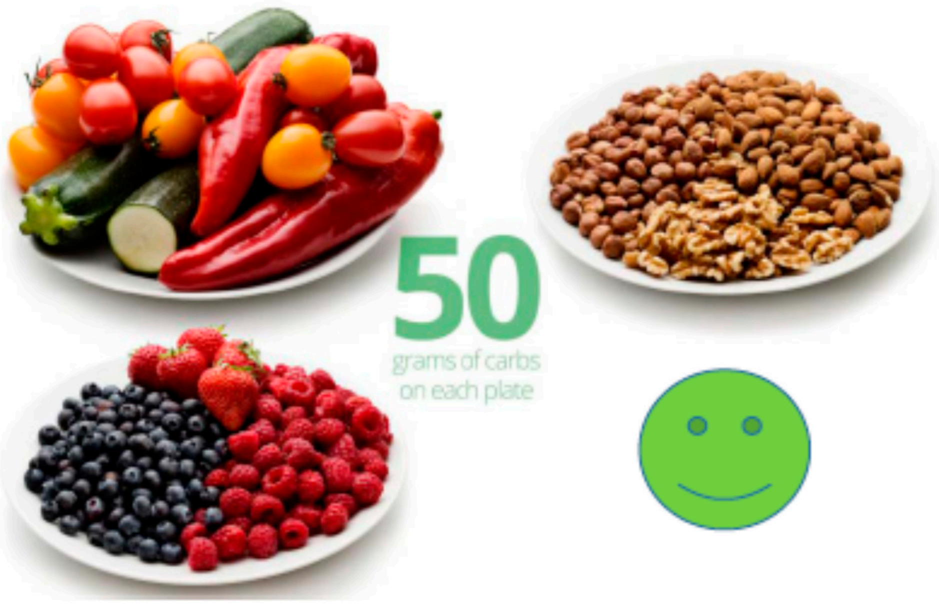

(Source: The Dietdoctor, 2017)

Please remember that the foods shown above are on the plates in isolation. Add to these the rest of the foods that you will consume throughout the day and all those carbs soon add up! The foods on the first picture will therefore be found on the list of food items that you will not be allowed to consume over the next eight weeks (see below).

What is fibre and why are we supposed to eat it?

Carbohydrates in our diet come from different sources, with some of them being more readily (if at all) absorbed by our bodies and used for energy to keep us going during the day. When nutritionists and other health professionals talk about carbohydrates and a certain type of carbohydrate diet (in this case "low carbohydrate diet") we mean carbohydrates that are actually available to our bodies. Only these types of carbohydrates are actually being counted when making recommendations for carbohydrate intake. The other type of carbohydrates, which are generally not available, are classed as dietary fibre (see below). There are also different subcategories of these, with some supplying our bodies with small amounts of energy, but these can be disregarded in the context of the foods that we consume every single day as part of a healthy balanced diet.

In the UK it is recommended that everyone in the UK above the age of 14 should aim to consume at least 30 grams of fibre per day. At the moment the majority of the population is not meeting these recommendations. Research has shown that sufficient fibre intake can help prevent heart disease, some cancers and diabetes. Fibre can also aid to improve your digestion and make you feel fuller for longer meaning you eat less and less often (serial snackers beware). Fibre can be found in a number of fruits, vegetables and nuts and seeds, i.e. plant-based foods.

Foods that contain 6 or more grams of fibre per 100g are classed as high-fibre foods, whereas those containing at least 3 grams of fibre per 100g are considered to be 'fibre-rich'.

Please ensure that if you think that your diet has been lacking in fibre so far to increase the amounts that you are consuming gradually over a couple of weeks and to make sure that you drink plenty of fluids. Otherwise you might have to deal with bloating and constipation as side effects.

Carbohydrate and fibre content of fruit and vegetables permitted on a low-carb diet

Food name	Amount available (digestible) carbohydrates in g (per 100g)	Amount fibre in g (per 100g)	Amount available (digestible) carbohydrates in g (per 80g portion)	Amount fibre in g (per 80g portion)
**Asparagus, boiled**	1.4	1.4	1.12	1.12
**Aubergines, fried**	2.8	2.3	2.24	1.84
**Avocado, average**	1.9	3.4	1.52	2.72
**Beans, green, boiled**	4.0	2.5	3.2	2.0
**Beans, soy/edamame**	5.1	6.1	4.08	4.88
**Broccoli, boiled**	2.8	2.3	2.24	1.84
**Broccoli, Purple sprouting, boiled**	1.3	2.3	1.04	1.84
**Brussel sprouts, boiled**	3.5	3.1	2.8	2.48
**Cabbage, green cooked**	2.3	2.6	1.84	2.08
**Cabbage, red cooked**	2.3	2.0	1.84	1.6
**Cabbage, white cooked**	3.2	1.4	2.56	1.12
**Cabbage, spring greens, cooked**	1.6	2.6	1.28	2.08
**Carrots, young, boiled**	2.3	1.84	4.4	3.52
**Cauliflower, cooked**	3.5	1.6	2.8	1.28
**Celery, raw**	0.9	1.1	0.72	0.88
**Coconut, desiccated**	6.4	13.7	5.12	10.96
**Coconut, fresh**	3.7	7.3	2.96	5.84
**Courgette, boiled**	2.0	1.2	1.6	0.96
**Cucumber, raw**	1.2	0.7	0.96	0.56
**Fennel, Florence, boiled**	1.5	2.3	1.2	1.84
**Kale, curly, boiled**	1.0	2.8	0.8	2.24
**Kohlrabi, boiled**	3.1	1.9	2.48	1.52

Food name	Amount available (digestible) carbohydrates in g (per 100g)	Amount fibre in g (per 100g)	Amount available (digestible) carbohydrates in g (per 80g portion)	Amount fibre in g (per 80g portion)
**Leeks, boiled**	2.6	1.7	2.08	1.36
**Lettuce, cos**	1.19	2.1	0.95	1.68
**Lettuce, iceberg**	1.77	1.2	1.42	0.96
**Lettuce, romaine**	1.19	2.1	0.95	1.68
**Mushrooms, oyster, raw**	3.79	2.3	3.03	1.84
**Mushrooms, Portobello, grilled**	2.24	2.2	1.79	1.76
**Mushrooms, white, boiled**	0.1	2.1	0	1.68
**Olives, green in brine, drained**	0.0	2.9	0	2.32
**Onions, fried (based on 2 table spoons, chopped)**	11.2	1.5	3.36	0.45
**Pepper, green, boiled**	2.6	1.8	2.08	1.44
**Pepper, red, boiled**	3.4	0.8	2.72	0.64
**Pepper, yellow, boiled**	5.3	0.8	4.24	0.64
**Pumpkin, boiled**	1.9	1.1	1.52	0.88
**Radishes, raw**	1.9	0.9	1.52	0.72
**Raspberries, red**	4.6	2.5	3.68	2.0
**Rocket**	0.0	1.3	0.0	1.04
**Spinach, baby, raw**	0.2	1.2	0.16	0.96
**Spinach, frozen, boiled**	0.5	2.1	0.4	1.68
**Spring onion, raw**	3.0	1.5	2.4	1.2
**Strawberries**	6.1	1.0	4.88	0.8
**Tomato, raw**	3.0	1.0	2.4	0.8
**Turnip, boiled**	2.0	1.9	1.6	1.52
**Watercress**	0.4	1.5	0.32	1.2

Carbohydrate and fibre content of nuts and seeds

A small handful (or about 30g) of nuts or seeds counts as a portion. Below some guidelines of how much fibre you would get from different types of nuts and seeds.

Food name	Amount available (digestible) carbohydrates in g (per 100g)	Amount fibre in g (per 100g)	Amount available (digestible) carbohydrates in g (per 30g portion)	Amount fibre in g (per 30g portion)
Almonds, raw	2.5	2.7	0.75	0.81
Brazil nuts	3.1	4.3	0.93	1.29
Chia seeds	6	38	1.8	11.4
Flaxseeds (Linseeds)	2	27	0.6	8.1
Hazelnuts	6.0	6.5	1.8	1.95
Pecan nuts	5.8	4.7	1.74	1.41
Pumpkin seeds	5.71	6.0	1.71	1.8
Walnuts	3.3	3.5	0.99	1.05

Permitted foods on a low-carb diet

Dietary fat – the star of the show? Following a high-fat diet

Cutting down on carbs to the extent that is necessary on a low-carb diet means that your energy needs to come from somewhere. Earlier on we already mentioned that fat can also be broken down into products (ketones) that supply our bodies with energy after a period of adaptation.

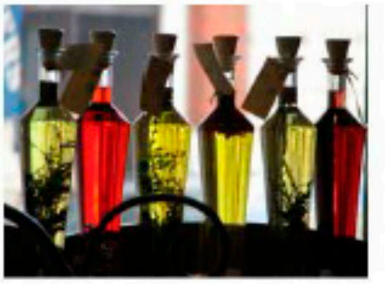

©creative commons 2016

Fat is also important role in our diet as it helps to absorb some vitamins that would otherwise simply go right through us. It also plays an important role in building the membranes of our cells. The key lies in the quality of the fats consumed with priority given to unsaturated fats, such as olive oil and other vegetable oils, avocados, nuts and oily fish. One type of unsaturated fat, which is essential to human health, are omega-3 fatty acids. These can be found both in animal and plant sources. Oily fish (see below) is an important source of omega-3 fatty acids as these are of the highest quality. However, it is possible for the body to convert the omega-3 fatty acids found in plants foods, such as nuts and seeds and their products, into the same end products found in oily fish. Coconut oil is also a fat recommended on a low-carb, high-fat diet and it is thought to be very healthy, despite a high proportion of saturated fats in it. Some people love to use it for everything, including frying their eggs. However, a small word of caution when it comes to taste as this is very distinct in coconut oil. For some individuals the egg-frying

method might be a bit too much for this reason. In other recipes, however, such as curries, soups and other dishes with lots of other ingredients, the taste of coconut oil complements and enhances the dish instead of tasting too overpowering.

Saturated fats have received a lot of bad press over the past few decades and whilst it is important to prioritise the fatty acids introduced above (mono- and polyunsaturated) a low-carb, high-fat diet doesn't place the same restrictions on saturated fats that you might have been used to so far. A lot of foods that contain a fairly high amount of saturated fats, such as meat, butter and cheese are actually encouraged to be consumed. You therefore don't need to worry about removing the visible fat from beef and pork steaks and other meats or about removing the skin from chicken. This will be one way of ensuring that you will achieve the amount of fat you will need to consume to replace the carbohydrates that you will omit from your diet. In addition, the marbling on cuts of meat will enhance the flavour whilst cooking it and also ensure that the meat stays moist during the cooking process.

One thing to avoid on a low-carb, high-fat diet are margarines as these are not a natural fat but have been manufactured and hardened from vegetable oils – a highly processed food. You should also not use vegetable oils that are high in omega-6 (as opposed to omega-3) fatty acids, such as sunflower oil, corn oil, soybean oil and cotton seed oil. Vegetable oils that should be consumed instead are coconut oil, olive oil and rapeseed oil

Dairy

Dairy products are both a source of fats and also proteins. Consuming a low-carb, high-fat diet means that you should not shy away from using full-fat versions of the product. Think butter, cream and jersey milk or at least full-fat milk. Stay away from low-fat yoghurts and also avoid fruit yoghurts as these contain too much sugar. Dairy is a good source of calcium and should therefore be included in our diet on a daily basis. However, you will also have to careful with the amount of milk that you are consuming as the milk sugar also contains glucose which will be absorbed by our bodies. A bit of milk (full-fat) in your tea and coffee is fine. However, a milkshake or hot chocolate should only be an occasional treat and then you still have to be mindful of the other ingredients.

Dairy products good to consume on a low-carb diet include full-fat versions of cheese, cream, cream cheese, crème fraiche, sour cream, quark and yoghurt.

Proteins to help you build and maintain that temple which is your body

Proteins are important in our diet as they have the vital functions of growth, maintenance and repair. They are also vital to help our immune system function properly. Proteins are composed of compounds called *amino acids* of which there are 20 that play a role in the human body. 8 of these amino acids are classed as essential because unlike the other 12 our bodies cannot produce these themselves, which means that they need to be obtained from our diets. This happens by consuming protein foods.

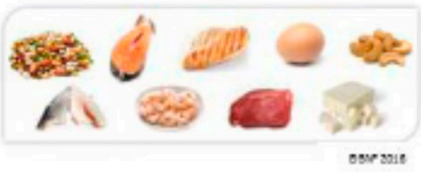

Whilst protein malnutrition can have disastrous consequences in reality the vast majority of people in the UK consume more than they actually need to.

The average UK male aged 15 to 64 should consume about 55 grams per day, the average UK female in this age group 45 grams per day. In order to achieve these recommendations we should eat two to three portions of protein each day. There are different types of protein sources permitted on a low-carb diet, including:

Animal-based proteins

Animal-based proteins contain all of the essential amino acids. This is because the animals have done the work for us eating a variety of food sources with different amino acid profiles and combing these in their muscles. The same is true for eggs where the egg yolk and the egg white have the role of sustaining the developing chick.

Meat

Meat is permitted on a low-carb diet as it contains virtually no carbohydrates. However, you should not eat more than 70g of processed meat per day, which includes sausages, bacon, cured meats (for example salami, chorizo) and reformed meats products (for example sliced packaged ham). Even on a low-carb diet consuming these foods in excess is not healthy as these not only tend to contain a number of additives but are also high in salt. Unfortunately, depending on the type of processed meat, the manufacturing process and the reputability of the manufacturer this might also mean that you might be consuming a low-quality product consisting of offcuts that will only be saleable if disguised in this form.

You should also be careful with deli products such as pates and hams (breaded is a no-no!) as these might contain hidden sugars, so it is best to check first before eating or even buying them.

Fish and seafood (Shellfish)

You should eat at least two portions of fish per week, one white, one oily. One portion is 140 grams which is about the size of a cheque book. The reason we recommend oily fish because these are an excellent source of omega-3 essential fatty acids, which we have mentioned earlier on. Fish also supply us with a number of vitamins and minerals. Remember that two portions per week is the minimum.

Please remember that you will not be able to eat battered or breaded fish whilst on low-carb!

White fish

Basra	Cod,	Coley	Dab	Flounder	Gurnard
Hake	Haddock	Plaice	Pollock	Red mullet	Tilapia

Some white fish should be eaten no more than once a week due to potentially high levels of pollutants contained in their flesh. These are seabream, seabass, halibut and turbot.

Oily fish

Anchovies	Carp	Herring	Kippers	Mackerel	Pilchard

Salmon	Sardines	Sprats	Trout	Tuna (fresh or frozen)	Whitebait

Swordfish should not be eaten more than once a week due to potentially high levels of pollutants. It should be avoided by children, pregnant women and those wanting to become pregnant.

Shellfish

Cockles	Crab	Langoustines	Mussels (and clams and winkles)
Oysters	Prawns	Scallops	Squid

It is important to note that processed, canned fish like tuna do no longer count as your 'oily' portion as the manufacturing process has taken all the omega-3 fatty acids out of them. However, the good news is – that tuna salad for your lunch still counts towards your portion of white fish.

One thing to bear in mind is choosing where possible sustainable sources of fish as recommended by the Marine Stewardship Council (MSC) in their *Good Fish Guide*. The MSC also run an accreditation scheme, so look out for certified products carrying the following logo:

Further information on the MSC and a regularly updated list of sustainable types of fish can be found at www.msc.org.

Eggs

Eggs are real powerhouses of nutrition and contain all the essential amino acids that humans need in their diet. One medium egg provides about 6.4grams of protein with the egg yolk containing higher concentrations of amino acids. However, as the egg white is larger than the egg yolk the majority of protein supplied when eating an egg will actually come from the egg white.

For animal welfare reasons we would recommend that you avoid buying the eggs from caged hens. However, the choice is absolutely yours.

A word on dairy products

Dairy products, such as milk, cheese, cream, sour cream and yoghurt, are also good sources of protein. However, as they also contain a fair amount of fat, we have included them in the fat section of this guide.

Plant-based proteins

Nuts and seeds are a great source of proteins and are also great sources of unsaturated fats, included important omega-3s.

Other plant-based protein sources include tofu, bean curd, mycoprotein (such as Quorn™) and soya-based meat replacement products, such as supermarkets' own brands. Please remember that the latter two are processed foods so it is always worth checking the label to ensure that there are not too many additives and flavour enhancers, salt and sugar found in

these products. If this is the case, you should either eat these products sparingly to ensure that you do not end up eating lots of hidden sugars by accident!

Beans and pulses, including lentils, although good sources of protein, are generally not recommended on a low-carb diet with the exception of green beans and soya beans as these don't contain as many carbs but are good sources of fibre.

Portion sizes for protein

Non-dairy

Eggs	2 medium-sized
Fish	140g (size of a cheque book) or 3 fish fingers
Meat, cooked	80g (size of a deck of cards or 3 slices)
Meat-replacement products	120g (for example two sausages or about the size of a snooker ball)
Nut butters, such as peanut butter	2 tablespoons (about the size of a golf ball)
Nuts and seeds	40g (1 small handful)

Vegetables

Vegetables growing above ground, for example artichokes, asparagus, aubergines, avocado, bok choi, broccoli, Brussel sprouts, cabbage, cauliflower, courgettes, cucumber, kale, lettuce, mushrooms (careful with shiitake mushrooms however, as they contain a lot more carbohydrates that other varieties!), olives, onions, peppers, spinach and other leafy greens, and tomatoes (Technically a fruit – we know ;-))

You have to be very careful with eating sweetcorn, corn on the cob and peas as these are higher in carbohydrates than other vegetables growing above ground and should only be eaten in moderation.

Fruit

As fruits contain a lot of carbs they should be eaten in moderation, only about 1 piece of fruit per day. Bananas and grapes are the worst offenders in a low-carb diet context. Berries can be eaten a bit more regularly but you should also be careful with these.

There are actually several reasons why eating vegetables and (some) fruits is good for you. Firstly, they contain a considerable amount of carbs in the form of fibre – the good stuff. Secondly, the different colours express the presence of different types of nutrients that are really good for us and are thought to prevent heart disease, diabetes and cancers. These are namely anti-oxidants and polyphenols. The different colours of the fruit and vegetables available for us mean that these contain different types of these nutrients – all-round protection.

If you feel slightly adverse to anything green and orange on your plate (one too many roast dinners with boiled-to-death cabbage) there are ways of incorporating these to help you feel less annoyed by their presence and to gradually get used to them. You could for example add some finely chopped carrots and vegetables to a tomato sauce with your fish or chicken.

Alternatively, you could make a mushroom pâté to create a delicious snack eaten with vegetable sticks.

Magnesium-rich foods

Magnesium plays an important role in our diet and obtaining it from a carefully planned low-carb diet is possible. However, there are some foods that will make it more likely that you will meet your daily requirements. These are artichokes, bone broth (we will give you a recipe for this), fish (highest are fresh tuna and halibut), nuts, spinach and other leafy greens.

Potassium-rich foods

Like magnesium, potassium is also important in a healthy, balanced diet and there are some foods that should be preferred to others in order to achieve your recommended intake. These are avocados, bone broth (again recipe to follow), cooked greens, cooked mushrooms, fish, meat and tomatoes.

Food no-no's on a low-carb diet

Sugar in any of its natural forms – This includes table sugar, brown sugar, demerara etc; honey, syrups in any form, molasses. Be aware of hidden sugars in food products and ready-meals. Later on in this guide we will show you how to read labels and identify these silent assassins that might jeopardise all your efforts.

If you can't do without sweetness for example in your tea or coffee there are some alternatives (in form of natural low-carb sweeteners and sugar alcohols) on the market that you can use sparingly. These are

- Stevia – a plant extract which is 200 to 300 times sweeter than sugar and has been used as a sweetener for many years in Asia and South America. Stevia is sold in UK supermarkets as Stevia sweetener and can also be found under the brand name "Truvia". However, be careful not to pick up a stevia-sugar blend by accident!
- Erythritol – a so-called sugar alcohol, which has about 70% of the sweetness of sugar but does not get broken down by our bodies (unlike sugar). Erythritol can be ordered online.

However, if you feel or experience that the sweet taste of these sweeteners induces cravings for sweet foods and carbs in general (which can happen in some cases) we would advise you to take the plunge and have your tea and coffee unsweetened.

As you will have probably expected chocolate is on the list of foods not to eat over the next 8 weeks. If you are really desperate you can have that odd piece of small (and we mean small!) piece of 70% or above dark chocolate, which contains about 3.5 grams of carbs per square. If you feel that you have to eat more than this small piece once you have started it is best to not eat it at all!

So what should you eat in a day if you are following a low-carb, high-fat diet

Following a low-carb, high-fat diet means that you can eat lots of permitted vegetables (preferably green as they are lower in carbs – but do incorporate some colour as well) and as many sources of high-quality fats as you like – avocado, butter, nuts, seeds and anything from the recommended dairy range. Fat has more calories per gram than carbs or proteins and will help you to feel full and satisfied quicker. Don't forget that if you have eaten a lot of carbs prior to embarking on this diet this might mean that it will take you a few days to adjust to your new style of eating and for your body to send the right signals that you are feeling full. Don't worry about this, after a few days chances are that you will eat less than you used to as you feel fuller sooner.

Fat will be the nutrient that you can eat as much as want of, however, this means only eating until you feel full and satisfied. If you go past the point of feeling full you will still overeat and undo all the goodness that you have done for your body. Listening to your body and knowing when to stop is the key!

One important thing to bear into consideration is to become more mindful when you are eating and to eat slowly, rather than devouring your food within 10 minutes because you feel that there is no time to eat. Eating in a rush and quite frankly 'shoving it in' means that by the time your brain has had the chance to signal your stomach that you have had enough food to satisfy all your physiological needs, thank you very much, you will have most likely overdone it already. You will be surprised to find that when you take it down a notch with the speed eating that you might want to eat far less than anticipated because before you know it you will feel full.

If your day really is that manic and lunch is a 10-minute affair or even takes place behind the wheel of your car (we live in busy, demanding times, we know, and sometimes your boss, children, the situation might not appreciate the eat slowly mantra) the good thing about a high-fat diet is that you can rest assured that once you feel full you will do so for longer. So if your lunch break is hectic eat slightly less at first. The feeling of fullness will come and if this is something that worries you, ensure that you have a snack handy that might fill that hole after all if you get the afternoon slump or your tummy starts rumbling again.

Do not eat more than 2 – 3 portions of protein from the non-dairy list per day. Remember, this is a high-fat diet, not a high-protein diet.

As you should not consume more than 50 grams of carbohydrates per day make sure that you do not eat any items on the 'no-no foods' list. Eating the recommended items from the vegetables and fruit list will help you get there, as will incorporating dairy products, nuts and seeds. This is not a no-carb but a low-carb diet so some carbs are necessary for you to consume and as we do not tend to eat every part of the animal in the UK in the same way as other cultures might do, which addresses their requirements for vitamins and minerals - think seal's eyeball as good sources of these (yum!) - it is important that you incorporate veggies and some fruit in your diet. We will give you a daily multi-vitamin and mineral supplement to

avoid any potential shortfalls, but this can only take you so far. The majority of your nutrients should come from your diet!

Suitable snacks

Overall, after a period of adjustment, we would expect for you not having to snack so much, if at all, as a low-carb, high-fat diet should keep you feel fuller for longer. However, do not despair there are some good snack options out there for you. It just won't be that piece of cake or a packet of crisps.

- Avocados – full of high-quality fats, creamy and delicious
- Canned mackerel in tomato sauce – just be careful with your breath with this one. And read the label to see if any sugars are lurking in that tomato sauce!
- Eggs (hardboiled) – ultimate, easy snack. Put a bit of salt on if you want!
- Slices of ham or salami and cheese rolled up and maybe some cheese spread on – think charcuterie in your favourite tapas restaurant. However, do remember to not eat more than 70 grams of these types of processed meats per day!
- Home-made low-carb crackers – hardly any carbs but lots of fibre. We will give you the recipe
- Nuts – again, pure natural goodness. But if you want some variation, we have some great and easy recipes on how you can flavour these.
- Olives – easy to get hold of with the added benefit of containing high-quality fats
- A piece of cheese – great source of calcium
- Pork scratchings – these can be a good alternative to that packet of crisps
- Some vegetables and a suitable dip – cucumber, celery, peppers, carrots. Again we have some great recipes for dips.

Some staples and alternatives to favourites on a very low-carb diet

- Almond flower – just because you are going low-carb does not mean that you cannot do some baking or have pancakes
- Bone broth – full of magnesium and potassium and other minerals to do your body good
- Bread – we will give you recipes for keto-bread if you can't be without.
- Coconut flower – Another low-carb alternative in cooking and baking
- Psyllium – To make keto crackers, porridge and increase fibre intake
- Pasta – Have spiralised vegetables, such as courgette instead. You can either make these yourself by investing in a spiralizer or they are now readily available from a number of supermarkets and discounters. They have all caught on to this trend.
- Porridge – A breakfast favourite of yours? Although oats are not allowed on low-carb there are some tasty alternatives, and we have the recipes to give you.
- Potatoes – Love mash? How about some caulimash instead? Or other vegetable mashes.
- Rice – A must when you are having curry? Or love risotto? Have cauliflower rice instead. You've guessed it – we do have some recipes for you.

Hydration

This is an important one that often gets overlooked. You should drink 6-8 glasses (200ml or medium-sized) per day to ensure staying hydrated. This will also help your body to cope with a potential increase in dietary fibre following a healthy diet (see above). However, you should stick to water, unsweetened black or herbal teas and black coffee or coffee with small amounts of full fat milk or cream. (We also have a recipe for *bullet proof* coffee.) Be careful with flavoured milks as these also tend to contain a lot of sugar which will catapult you right out of the max. 50g of carbs per day zone! If you think that plain water is too boring there are some ways (and recipes) to make this more interesting, such as adding slices of citrus fruit and/or mint or cucumber for example. If you are struggling with the taste of tea and coffee without sugar there are some alternatives on the market that you can use sparingly. These can be found in the section about sugar on page 21.

You should also not drink any fruit juices as the natural sugar contained in fruits has been freed through the process of liquidisation. This means it is more easily digested and can raise your blood sugar a lot quicker. The fruit sugar contained in fruit (fructose) can actually not be metabolised by our bodies and if not turned into energy will be converted into body fat. In addition, this free sugar can contribute to dental decay.

Alcohol

Small glasses of dry wine are permitted and these contain about 2 grams of carb per glass. So enjoy your glass of wine, but do not overdo it! Spirits such as whisky, brandy, vodka, sugar-free cocktails are also permitted. But be careful not to mix these with soda as this will make your drink too sugary. Beer (think liquid bread!), cider and mixer drinks (even that G&T) with sugary sodas or alcopops are not allowed as these contain far too many carbohydrates, which will be broken down fairly quickly and end up in your blood stream and cells. Bye-bye low-carb diet! Check the labels of diet versions of diet sodas to see whether they contain 0 sugars/carbs and these might be an alternative. However, do bear in mind what we said about artificial sweeteners and their potential to still induce sugar cravings. Furthermore, there is currently debate on whether some artificial sweeteners still elevate your insulin levels. We therefore strongly advise you to proceed with caution here at least whilst you are part of our cohort.

Learning to read labels and nutritional information

This is an important one when food shopping and eating out in places that provide you with information on the nutritional content of their dishes, for example fast food restaurants and pubs. If you are unsure check out the label for the carbohydrate content of the food. Avoid anything with more than 5gram per 100 grams of carbs or more than 5 % of carbs.

Label reading

In the UK food labels can be found at the back and depending on the manufacturer or retailer also at the front of the packaging.

Labels at the back of the packaging

Below is the back of pack nutrition label for Heinz Tomato soup as an example.

Ea

	Per 100g	Per 1/2 can	%RI*
Energy	215kJ	429kJ	-
	51kcal	102kcal	5%
Fat	2.1g	4.3g	6%
-of which saturates	0.2g	0.4g	2%
Carbohydrate	6.8g	13.6g	5%
-of which sugars	4.8g	9.7g	11%
Fibre	0.6g	1.3g	-
Protein	0.8g	1.7g	3%
Salt	0.5g	1.1g	18%

*RI per serving. Reference intake of an average adult (8400kJ/2000kcal)

Please note that you will probably eat more than ½ can!

This means a full can contains 27.2g of carbs, which is more than half your daily allowance!

Please note that this is based on the average UK woman

Eating a full can of soup will provide you with 2.6g of fibre

There are a few things to be aware of:

The manufacturer's portion size guide might actually not be a realistic reflection of what you might actually eat yourself and it can be very easy to underestimate the amount of carbohydrates you are consuming.

Another thing to take into consideration is the quality of carbohydrates you are eating. The label will not tell what source the total sugars contained in foods derive from and can be a combination of natural sugars found in fruits (fructose) and milk (lactose) where applicable and added sugars.

This is where looking at the ingredients list (also a the back of pack) might necessary as sugar comes under many different names and there might be some surprises in store for you.

As a general rule, the closer an added sugar is towards the top of the ingredients list the more of it will be contained in the product. There might also be different types of added sugar in the same product.

All of these that you might spot in the ingredients list are actually added sugars

> agave sugar, brown sugar, cane sugar, dextrose, fructose, fruit juice concentrate, glucose, golden syrup, HFCS/high fructose corn syrup, honey, hydrolysed starch, invert sugar, isoglucose, levulose, maltose, modified starch molasses, sucrose, syrups (sucrose, glucose, malt, corn, maple), treacle,

Once a product crosses specific thresholds for specific nutrients it will either be classed as 'medium' or 'high' in sugar or other nutrients (see below).

	Low	Medium	High
**Total sugars**	5g per 100g product (or 5%)	More than 5g but less than 22.5 per 100g product (between 5% and 22.5%)	More than 22.5g per 100g product (or 22.5%)
**Salt**	0.3g of salt or less per 100g (or 0.1g sodium)	More than 0.3g of salt (0.1g sodium) but less than 1.5g of salt (0.6g sodium) per 100g product	More than 1.5g of salt per 100g (or 0.6g sodium)

If the portion size means that more than 25g of sugar would be consumed with one portion the product is automatically classed as 'high sugar'.

For drinks containing total sugar the rule is that 2.5g per 100ml is classed as 'low', between 2.5 and 11.25g per 100ml is classed as 'medium' and above 11.25g per 100ml is 'high'. If the

portion size of a drink means that more 13.5g of sugar would be consumed the product is automatically classed as 'high sugar'.

Front of pack labels

Some retailers and manufacturers display a food label at the front of the pack highlighting whether some of the crucial nutrients are contained in the foods to low, medium or high levels. This is called the 'traffic light system'. The main nutrient that you will need to look out for is the 'sugars' category. You do not need to concern yourself with the 'fat' and 'saturates' categories on a low-carb diet as the permitted amounts are different from the standards applied to the traffic light labels. These are based on the UK dietary guidelines.

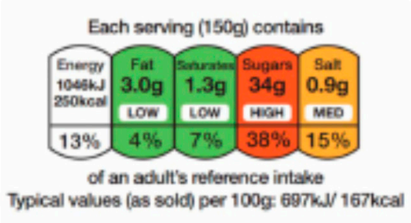

Interpreting nutritional information provided by fast food restaurant and pub chains

A number of high street fast food outlets and pub chains publish nutritional information on typical serving sizes of their dishes that you can access online, download or on request at the restaurant.

Being aware of the nutritional content of some of these dishes is helpful if you tend to eat out more often as it can be difficult in this case to stick to a low-carb diet and you might unknowingly jeopardise all your efforts.

It is also great to make you aware of how much individual components will add up. Watch out for hidden carbs, including starches and sugars in sauces, salad dressings and coatings.

Some example nutrition guides are attached to this guide to give you an idea what to look out for. However, these are by no means meant as endorsements of particular eateries.

Tips for Eating Out on a low-carb, high-fat diet

Eating out whilst on a low-carb diet can be quite straight forward depending on the type of cuisine that you choose and very often it is easy to make your meal low-carb friendly. We have also provided you with example nutritional information from some of the pub and restaurant chains in the UK to give you examples what to look out for. In general, all chains should have this information readily available for interested diners in the restaurants and on their websites. If you would like us to try and find the nutritional information for a particular place, please do let us know.

- Many restaurants and pubs now will allow you to swap your potatoes and chips with a side salad.
- If you fancy a burger just have it without the bun or if you feel comfortable doing so ask for it to be wrapped in a large lettuce leaf instead (so you can still pick it up)

- Instead of breaded/battered fish or chicken have the steamed or roasted versions instead.
- If you get a Mexican or a Subway takeaway ask for the salad version of the dish rather than the wrap/burrito.
- Be careful with sauces and gravies as these might contain flour (and carbs!). In order to control how much (and if) you want to eat any of this ask for it to be served on the side rather than on your plate.
- If you fear that you might feel a bit hungry still after you have eliminated the starchy foods from your restaurant plate, ask for (extra) butter or olive oil to make up for this. Some people following a low-carb take a small bottle of olive oil with them when eating out just in case.
- If crave a third course see if there is a cheese platter on the menu (without the crackers!) instead of opting for pudding.
- It might be difficult to eat in Indian or Chinese buffet restaurants or takeaways whilst you are participating in the CALIBER study. Other research undertaken by our nutrition team has shown these dishes to be very high in added sugars. You definitely need to avoid the sweet and sour chicken! However, Indian creamy curries and kebabs might be a good option.
- Go easy on the condiments as ketchup, cocktail and BBQ sauces can contain a lot of added sugar.
- If you know that you have been invited to a dinner party and you don't want to offend, be careful of you carb intake earlier during the day. This way you can at least try and contain some of the damage.
- Otherwise, if your host is understanding give them a fair warning, which should be much appreciated. Some low-carbers excuse their avoidance of starchy foods with stomach issues. If you think that you might not be able to eat enough at the dinner party, have a snack at home before you leave.
- Pizza is a harder one! It should be avoided during your eight weeks on our study. If you really crave pizza, you can use your own using an alternative base. Please see the recipe that we have provided for this.

Appendix - Helpful App to help you to stick to a low-carb diet

Change4Life – Sugar smart App

Change4Life is a public health initiative run by Public Health England. This app lets you scan the barcodes of about 87,000 food products available from UK major manufacturers and retailers. It focuses on the amount of free sugar in these foods and uses a traffic light system to let you know whether these would be high in the nutrients. A good way to find out about any unexpected sugars in products that you might want to buy.

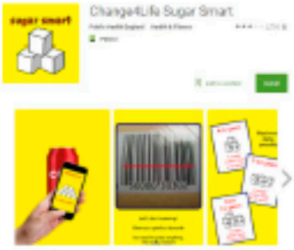

The app can be downloaded for free from the Appstore and GooglePlay.

Appendix - Examples nutritional information fast food, pub and restaurant chains

ASK

Italian

Recipe / Dish	Menu selections	Per 100g** Energy KJ	Kcal	Fat (g)	Saturates (g)	Carbs (g)	Sugars (g)	Protein (g)	Salt (g)	Per Serving** Energy KJ	Kcal	Fat (g)	Saturates (g)	Carbs (g)
Italian Olives	Breads / Nibbles	673	163	15	2.0	3.8	0.5	1.0	3.0	841	204	19	2.5	4.8
Rosemary & Sea Salt Bread	Breads / Nibbles	1049	251	5.7	0.8	41	2.8	7	1.1	2089	499	11	1.5	81
Garlic Bread Speciale	Breads / Nibbles	1134	270	11	6.4	33	2.4	11	1.1	3069	729	29	17	69
Garlic Bread	Breads / Nibbles	1144	274	7.8	1.9	38	2.6	8.8	1.1	2455	588	17	4.1	82
Garlic Bread with Mozzarella	Breads / Nibbles	1153	276	11	4.8	29	2.2	11	1.2	3281	784	31	13	83
Cheese Fonduta	Breads / Nibbles	1121	264	11	4.2	33	1.2	6.7	1.2	3268	771	33	12	96
Antipasti - The Warm One	Starters & Shares	934	223	13	3.3	17	1.5	6.8	0.8	5946	1422	83	21	109
Antipasti - The Cheese One	Starters & Shares	1143	274	17	7.9	22	4.7	6.9	0.8	5797	1388	85	40	111
Antipasti - The Meat One	Starters & Shares	1123	268	16	3.4	22	1.0	10	1.7	4778	1140	70	15	92
Antipasti - The Mixed One	Starters & Shares	1181	284	16	7.2	21	4.5	9.5	1.0	6288	1508	98	38	111
Mussel Marinara	Starters & Shares	448	106	6.4	0.6	7.9	1.6	3.9	0.6	1564	371	22	2.1	[illegible]
Bruschetta	Starters & Shares	692	165	8.5	1.6	18	2.0	4.5	1.0	1343	321	17	3.2	34
Calamari (1 Serving)	Starters & Shares	843	201	14	1.5	11	0.9	7.3	1.0	1507	360	25	2.8	19
Mushrooms Al Forno	Starters & Shares	1291	330	27	5.3	10	1.3	6.3	0.6	2274	540	43	6.6	29
Meatballs	Starters & Shares	815	195	9.7	3.5	16	2.1	12	1.4	1223	292	15	5.3	24
Insalata Caprese	Starters & Shares	776	187	15	7.0	1.6	1.6	11	0.5	1622	391	32	15	3.4
Crocchette	Starters & Shares	888	213	14	3.9	14	1.4	6.8	[illegible]	1768	423	26	5.7	29
Butterfly King Prawns	Starters & Shares	923	221	11	3.4	14	1.7	6.3	1.0	1737	416	21	6.3	27
Melanzane al Forno	Starters & Shares	631	151	12	3.6	5	2.5	5.8	0.6	1259	301	24	7.3	10
Tagliatelle Carbonara	Fresh Pasta	497	232	15	8.1	15	0.9	9.8	0.9	4535	1079	69	39	70
Aragosta e Gamberoni	Fresh Pasta	628	149	4.9	1.8	17	2.0	8.5	1.0	2798	652	21	8.1	75
Beef Tortellini	Fresh Pasta	513	123	5.5	2.8	13	2.0	6.4	0.9	1619	387	18	8.9	39
Funghi Misti	Fresh Pasta	596	142	72	4.1	18	3.4	4.7	0.4	2588	618	33	18	76
Rigatoni con Zucchine e Pesto - without chicken	Pasta - Classic	1112	[illegible]	[illegible]	8.4	27	2.8	7.1	1.0	[illegible]	[illegible]	[illegible]	[illegible]	91
Fettuccine Bolognese	Pasta - Classic	604	138	5.9	1.1	25	3.1	6.6	[illegible]	2925	692	21	4.6	97
Spaghetti al Pomodoro	Pasta - Classic	[illegible]	129	5.8	1.6	30	2.7	4.3	[illegible]	2815	672	24	7.6	98
Linguine Carbonara	Pasta - Classic	1209	287	16	9.0	38	3.0	5.5	[illegible]	4054	1035	58	32	103
Linguine con Frutti di Mare	Pasta - Classic	[illegible]	187	4.8	0.9	28	1.6	7.2	1.9	2977	708	22	2.4	89
Ravioli Marittimi	Pasta - Classic	721	170	7.9	4.0	28	[illegible]	7.6	[illegible]	2946	411	28	11	68
Rigatoni di Manzo Piccante	Al Forno	720	171	9.1	3.0	15	2.1	9.2	[illegible]	2075	545	45	17	85
Penne al Pollo della Casa	Al Forno	634	151	7.3	3.7	14	1.6	7.7	[illegible]	2625	637	41	21	76
Lasagne	Al Forno	692	166	10	4.0	11	2.6	6.4	[illegible]	3096	742	47	15	51
Melanzane al Forno - Without Side Salad	Al Forno	631	151	11	3.6	5	2.5	5.8	[illegible]	2547	609	49	14	20
Risotto Pescatore	Risotto	[illegible]	[illegible]	[illegible]	1.1	19	1.1	5.9	[illegible]	[illegible]	812	28	6.8	128
Risotto con Pollo e Funghi	Risotto	579	139	4.0	2.0	19	1.2	5.7	1.1	3420	818	24	12	114
Risotto Rosso	Risotto	588	139	5.0	2.5	20	1.7	6.5	1.0	3333	797	29	14	116
Pork Belly Porchetta	Meat & Fish	815	196	15	5.5	5.8	2.6	10	[illegible]	4873	1143	87	32	[illegible]
Pollo Prosciutto	Meat & Fish	474	112	4.5	1.9	7.0	1.0	12	0.8	2378	562	22	9.5	35
Sea Bass al Forno	Meat & Fish	481	[illegible]	[illegible]	2.4	5.7	[illegible]	6.4	[illegible]	2335	560	29	13	31
Prima Pizza Salami Misti	Pizza & Calzone	856	203	8.9	4	21	2.3	11	1.2	4255	1011	44	30	103

Product/ Dish Description	Nutrition Information Per Portion							
	kJ	kcal	Fat (g)	Saturates (g)	Carbohydrate (g)	Sugars (g)	Protein (g)	Salt (g)
**DAYTIME VALUE & SNACKS MENU**								
**STARTERS**								
TOMATO SOUP	980	234	7.7	3.3	33.0	11.8	5.7	1.8
CRISPY POTATO DIPPERS	2045	488	28.6	12.1	34.0	2.7	23.6	1.7
BUBBLE & SQUEAK	1458	348	24.4	10.9	19.5	3.4	10.4	1.4
GARLIC & HERB BREADED MUSHROOMS	1627	389	17.3	1.9	53.0	6.7	8.7	1.5
**MAINS**								
SMOTHERED CHICKEN	3991	953	45.3	15.1	79.0	11.9	55.4	4.5
LASAGNE	2653	634	28.1	12.1	64.3	17.0	27.9	3.1
GRILLED GAMMON STEAK WITH EGGS	3841	917	37.8	12.5	62.5	5.6	80.1	4.5
GRILLED GAMMON STEAK WITH PINEAPPLE	3655	873	31.3	10.9	81.2	26.4	67.8	4.2
GRILLED GAMMON STEAK WITH ONE OF EACH	3748	895	34.6	11.6	71.9	16.0	74.0	4.3
FISH & CHIPS WITH PEAS	5227	1248	73.2	12.3	107.4	6.9	37.5	1.7
FISH & CHIPS WITH MUSHY PEAS	5386	1286	73.3	12.4	113.9	5.8	39.8	2.6
BATTERED GIANT HADDOCK & CHIPS WITH PEAS	4501	1075	59.3	16.2	86.3	7.7	47.4	1.7
BATTERED GIANT HADDOCK & CHIPS WITH MUSHY PEAS	4660	1113	59.4	16.3	92.8	6.4	49.7	2.6
MEXICAN BEEF CHILLI	2999	716	18.6	5.4	97.1	5.7	28.7	2.2
THREE CHEESE CRUSTLESS QUICHE	2390	571	36.4	18.0	42.2	10.6	19.7	1.2
THE SOUTH WESTERN BURGER	4105	980	47.6	11.0	106.5	12.4	29.6	2.4
BEEF, CHEESE & MUSHROOM BURGER	4846	1157	64.5	23.0	93.3	12.3	48.7	2.5
GRILLED CHICKEN & BACON SALAD	1816	434	19.0	5.6	12.4	11.3	50.9	4.7
CHICKEN TIKKA CURRY	3614	863	24.0	6.2	113.1	25.6	45.3	3.7
BREADED WHOLETAIL SCAMPI WITH PEAS	3885	928	44.7	8.3	107.0	7.0	24.2	3.9
BREADED WHOLETAIL SCAMPI WITH MUSHY PEAS	4044	966	44.8	8.4	113.6	5.7	26.5	4.8
SMOKY PAPRIKA CHICKEN	1802	430	14.7	4.1	31.9	19.1	41.4	2.2
SAUSAGE, EGG & CHIPS	4170	996	57.1	18.9	74.2	7.4	44.0	2.5
SWEET POTATO & FETA LASAGNE	3106	742	39.1	15.8	69.9	17.8	24.2	3.0
HOT 'N' SPICY VEGGIE NACHO BURGER	5160	1233	61.8	19.9	142.3	17.9	23.0	3.0
HAM & CHEESE SANDWICH WITH WHITE BREAD	2613	624	28.3	12.6	55.8	3.0	35.2	3.5

Five Guys

Typical values per portion

	Energy, kJ	Energy, kcal	Total fat, g	Of which Saturated, g	Carbohydrates, g	of which Sugars, g	Fibre, g	Protein, g	Salt, g	Average portion size (g)
**MEAT**										
Bacon	267.2	64.5	5.40	2.2	0.2	0.0	0.1	3.9	0.97	11.3
Beef Burger Patty	745.4	179.0	13	5.1	0.1	0.1	0.3	16	0.12	61.5
Hot Dog	974.2	235.0	20	8.5	0.5	0.7	0.4	13	1.49	88
**BUN**										
Burger bun	959.4	227.7	7.20	2.48	38	5.83	1.34	5.78	0.47	73.8
Hot dog bun	1067.8	253.2	7.90	2.66	40	6.99	1.42	6.26	0.55	82.3
**FRIES**										
Little FRIES - COOKED IN PEANUT OIL	2980.2	716.0	44	5.81	72	0.79	7.66	11	1.14	284.2
Regular FRIES - COOKED IN PEANUT OIL	4823.3	1158.8	72	9.41	118	1.28	12	18	1.85	427.8
Large FRIES - COOKED IN PEANUT OIL	7178.8	1724.8	107	14	173	1.81	18	28	2.75	838.4
Cajun Seasoning	51.0	12.2	0.20	0.03	1.77	0.65	0.65	0.49	0.70	4.25
**TOPPINGS**										
BBQ Sauce	86.3	20.4	0.08	0.02	4.11	3.95	0.39	0.23	0.35	15
Cheese (1 Slice)	300.9	71.8	5.81	3.72	0.58	0.58	0.00	3.78	0.72	19.4
Green Peppers	8.3	1.9	0.02	0.01	0.43	0.22	0.16	0.08	0.00	9.25
Grilled Mushrooms	51.2	12.1	0.13	0.03	1.97	0.03	0.75	1.14	0.01	32.8
Hot Sauce	8.3	1.9	0.05	0.01	0.14	0.02	0.12	0.19	0.65	7.75
HP Brown Sauce	63.3	12.5	0.01	0.01	3.47	2.83	n/a	0.11	0.16	12.3
Jalapeno Peppers	4.4	1.0	0.03	0.01	0.56	0.33	0.22	0.07	0.02	8
Tomato Ketchup	72.9	17.1	0.02	0.00	3.89	3.82	n/a	0.20	0.30	18.8
Lettuce	8.8	2.9	0.02	0.00	0.27	0.00	0.18	0.14	0.00	15
Mayonnaise	441.2	107.3	12	0.88	0.49	0.49	n/a	0.13	0.24	18.3
Mustard	19.4	4.7	0.24	0.05	0.27	0.05	0.17	0.27	0.17	6.3
Onions	31.9	7.0	0.02	0.00	1.58	0.74	0.30	0.19	0.00	17.5
Grilled Onions	45.2	10.8	0.11	0.03	2.47	1.48	0.80	0.37	0.00	28.8
Pickles	9.0	1.9	0.00	0.00	0.27	0.24	0.38	0.21	0.57	23.8
Relish	104.5	24.8	0.06	0.03	5.62	4.64	0.19	0.06	0.22	18
Tomatoes	24.8	5.8	0.04	0.00	1.21	1.21	0.40	0.04	0.00	40.3

Watch out when you are adding extras to your burger

Harvester

STARTERS AND SHARERS	Energy (KJ)	Energy (Kcal)	Fat (g)	Saturated Fat (g)	Carbohydrate (g)	Sugars (g)	Protein (g)	Salt (g)
Potato Skins with Bacon & Cheese	1,934	460	26.2	13.5	32.2	2.1	22.8	2.15
BBQ Chicken Wings	1,593	379	18.4	4.8	20.3	18.6	32.8	1.98
Crispy Coated Chicken Bites	1,683	401	17.9	3.2	36.9	18.3	22.9	2.21
Cheese Popits with Salsa V	1,759	419	25.9	10.1	33.1	4.4	12.2	1.46
Spicy Crackerjack King Prawns	1,359	324	13.3	3.1	40.0	13.6	9.3	2.02
Breaded Mushrooms V	2,363	563	39.2	6.0	43.6	2.2	8.1	1.88
Chicken & Chorizo skewers NEW	1,990	474	36.5	12.4	6.7	5.5	28.3	1.81
Ultimate Nachos V	3,633	865	48.7	15.8	82.0	4.8	20.3	3.03
Why not add Three Bean Chilli V	464	110	3.0	0.4	15.1	6.8	4.2	0.78
Or add BBQ Pulled Pork	1,298	309	10.1	3.3	40.4	38.7	13.6	1.43
Creamy Tomato & Basil Soup V NEW	1,268	302	5.7	2.7	53.5	4.9	8.0	2.26
Sticky Duck Wings	1,839	438	19.1	5.1	35.5	32.5	29.2	2.69
Fish Basket NEW	2,994	713	45.2	7.5	54.5	7.5	21.7	4.00
Cheesy Garlic Bread Board V NEW	5,795	1,380	92.6	31.8	108.4	15.3	34.0	5.15
**CHICKEN**	**Energy (KJ)**	**Energy (Kcal)**	**Fat (g)**	**Saturated Fat (g)**	**Carbohydrate (g)**	**Sugars (g)**	**Protein (g)**	**Salt (g)**
Harvester's Famous 1/2 Rotisserie Chicken	1,885	449	22.9	5.0	2.4	3.5	57.1	1.58
Whole Rotisserie Chicken NEW	3,525	839	41.2	10.0	2.4	4.0	113.5	3.07
Chargrilled Chicken Breast	1,126	268	9.1	1.0	2.5	3.1	43.0	1.48
Triple Chicken	2,763	658	27.7	3.7	13.6	3.6	87.3	2.94
Chicken Skewer	1,879	447	25.9	6.0	2.5	3.3	49.7	0.92
Salsa Chicken & Pepper Stack	1,588	378	14.0	1.2	11.4	11.4	49.0	1.90
HARVESTER RECOMMENDS BBQ Brushed & Basted	4,487	1,068	43.8	9.3	103.9	47.4	63.0	3.67
HARVESTER RECOMMENDS Piri Piri Brushed & Basted	3,952	941	48.0	9.6	63.8	8.0	62.1	6.22
Garlic & Parsley Brushed & Basted	6,259	1,490	40.3	12.9	59.9	73.1	69.8	3.1
Farmer Rikki's Hot Chilli Sauce Brushed & Basted	4,117	980	52.0	9.9	62.7	5.8	63.1	2.99

CALIBER Menu 1 (DK18) – Low-carb, high-fat – Week 1

	Day 1	Day 2	Day 3	Day 4	Day 5	Day 6	Day 7
**Breakfast**	Simply eggs and avocado<br>Coffee (no sugar) with a little full-fat milk	Greek yoghurt, mixed berries and chia seeds<br>Coffee (no sugar) with a little full-fat milk	Judy's fabulous oatmeal<br>Coffee (no sugar) with a little full-fat milk	Greek yoghurt, mixed nuts and chia seeds<br>Coffee (no sugar) with a little full-fat milk	Judy's fabulous oatmeal<br>Coffee (no sugar) with a little full-fat milk	Grilled parmesan tomatoes<br>Coffee (no sugar) with a little full-fat milk	1 sausage, mushrooms, tomatoes, avocado<br>Coffee (no sugar) with a little full-fat milk
**Lunch**	Cheese and herb muffins<br>Cherry tomatoes, snack cucumbers	Bacon-wrapped haloumi<br>Cheese and herb muffins	(Left-over) Courgetti Bolognese	Greek salad with walnuts and avocado	lChef's salad bowl	Left-over butter chicken curry with cauli-rice	Roast lamb with summer vegetables and mint pesto
**Dinner**	Rosemary salted sirloin with lemon and avocado butter, fried mushrooms and steamed broccoli	Courgetti Bolognese with parmesan cheese	Fish stew with dill and prawns, cauli-rice and steamed broccoli	Stir-fried pork with watercress<br>Cherry tomato salad with balsamic vinegar and olive oil	Creamy butter chicken curry with cauli-rice	Meatloaf with courgette and garlic gratin and heavenly sauce	Cauli-pizza with ham, salami and olives
**Snacks**	Devilled nuts,<br>1 bag of Pork scratchings,<br>2 handfuls of strawberries	2 x mini baby bel<br>1 handful of walnuts	Vegetable sticks (red peppers, carrot sticks, cucumber) with mushroom pate	Salami, cheese and cucumber rolls<br>1 handful of almonds<br>2 handful of raspberries	Avocado,<br>1 handful of olives	1/2 tub of olives with cheese, nutty crackers with (left-over) mushroom pate	2x mini baby bel, vegetable sticks, 1 handful of walnuts
**Drinks**	Bullet-proof coffee, Coffee (no sugar) with a little full-fat milk or cream; Tea (no sugar) with a little full-fat milk or unsweetened herbal teas; Water (unflavoured or flavoured with citrus fruit, mint)						

Table S2. Per Protocol analysis of changes in anthropometric variables and biochemical markers of metabolic health following the LCHF and HCLF diets for 8 weeks.

Measurement	VLC Week 0	VLC Week 4	VLC Week 8	HC Week 0	HC Week 4	HC Week 8	TIME	T X G
Mass (kg)	91.66 ± 14.66	88.41 ± 14.57^{bb}	86.39 ± 14.25 ^{bbb}	72.48 ± 14.72	72.01 ± 14.88	72.27 ± 14.7	<0.001	<0.001
BMI (kg/m^2)	29.34 ± 1.45	28.27 ± 1.45^{bb}	27.61 ± 1.26^{bb}	24.56 ± 3.83	24.40 ± 3.98	24.50 ± 3.98	<0.001	<0.001
FM (kg)	32.72 ± 4.19	30.16 ± 3.73^{bb}	29.10 ± 3.41^{bb}	19.89 ± 7.46	19.48 ± 7.89	19.60 ± 8.22	<0.001	<0.001
FFM (kg)	58.87 ± 11.71	58.25 ± 11.92^{e}	57.28 ± 11.95^{b}	52.59 ± 11.63	52.52 ± 11.74	52.67 ± 11.54	0.009	0.003
SBP (mmHg)	131 ± 10	120 ± 11^{b}	123 ± 9^{bb}	127 ± 12	128 ± 12	129 ± 14	0.035	0.009
DBP (mmHg)	84 ± 9	77 ± 10^{b}	75 ± 9^{bbb}	78 ± 8	79 ± 8	78 ± 9	0.003	0.004
Glucose (mmol/L)	5.72 ± 0.44	5.83 ± 0.48	5.68 ± 0.47	5.79 ± 0.35	5.60 ± 0.54	5.80 ± 0.53	0.919	0.142
Insulin (pmol/L)	65.41 ± 29.35	55.04 ± 13.71	41.13 ± 17.40	60.26 ± 15.48	52.44 ± 8.42	44.02 ± 11.23	0.004	0.723
rQUICKI	0.35 ± 0.03	0.34 ± 0.34	0.37 ± 0.37	0.35 ± 0.03	0.36 ± 0.02	0.38 ± 0.04	0.003	0.703
HOMA IR	2.81 ± 1.36	2.4 ± 0.63	1.79 ± 0.79	2.51 ± 0.64	2.19 ± 0.48	1.94 ± 0.63	0.013	0.600
Cystatin C (ug/ml)	0.38 ± 0.12	0.32 ± 0.07	0.36 ± 0.09	0.41 ± 0.13	0.43 ± 0.17	0.43 ± 0.15	0.608	0.183
Ferritin (ng/ml)	186.27 ± 176.78	175.11 ± 168.09	173.14 ± 162.87	155.38 ± 83.3	128.14 ± 72.66	141.38 ± 89.82	0.019	0.355
NEFA (mmol/L)	0.84 ± 0.32	1.00 ± 0.30	0.80 ± 0.24	0.76 ± 0.25	0.79 ± 0.22	0.62 ± 0.17	0.083	0.691

Values are expressed as means ± SD of n=8 VLC & n=8 HC. Non-parametric testing values are expressed as median ± IQR of n=7 VLC & n=8 HC. ^{b} *P < 0.05,* ^{bb} *P < 0.01,* ^{bbb} *P < 0.001 denotes significantly different to baseline,* ^{e} *P < 0.05, denotes significantly different to endpoint *P < 0.05 denotes significantly different between groups at that timepoint. DBP, Diastolic blood pressure; FM, Fat Mass; FFM, Fat-free mass; HOMA IR, homeostatic model of insulin resistance; NEFA, non-esterified fatty acids; rQUICKI, revised Quantitative Insulin sensitivity Check Index;* SBP, systolic blood pressure.

Table S3. ANCOVA analysis using baseline BMI as a covariate on cardiometabolic health markers following the LCHF and HCLF diets for 8 weeks.

	LCHF			HCLF					
**Measure**	**Week 0**	**Week 4**	**Week 8**	**Week 0**	**Week 4**	**Week 8**	**TIME**	**T X G**	**Group**
SBP (mmHg)	125 ± 4	114 ± 4	114 ± 3	133 ± 4	134 ± 4	136 ± 14	0.344	0.015	0.080
DBP (mmHg)	79 ± 3	73 ± 4	70 ± 3[bb]	82 ± 8	82 ± 8	82 ± 3	0.470	0.038	0.135
Insulin (pmol/L)	63.44 ± 10.60	52.24 ± 4.96[b]	37.50 ± 6.39[b]	61.98 ± 9.74	54.88 ± 4.56[b]	47.20 ± 5.87[b]	0.767	0.725	0.680
HOMA IR	2.64 ± 0.47	2.20 ± 0.23	1.58 ± 0.31[b]	2.67 ±0.43	2.36 ± 0.21	2.12 ± 0.28[b]	0.889	0.714	0.543
rQUICKI	0.35 ± 0.12	0.34 ± 0.07[b]	0.39 ± 0.15[b]	0.34 ± 0.11	0.35 ± 0.06[b]	0.37 ± 0.14[b]	0.363	0.561	0.661
Glucose (mmol/L)	5.57 ± 0.16	5.59 ± 0.20	5.46 ± 0.20	5.91 ± 0.15	5.81 ± 0.18	5.99 ± 0.19	0.465	0.441	0.173
Cystatin C (ug/ml)	0.32 ± 0.05	0.26 ± 0.05	0.30 ± 0.05	0.46 ± 0.05	0.48 ± 0.05	0.48 ± 0.05	0.950	0.341	0.029
NEFA (mmol/)	0.81 ± 0.13	1.01 ± 0.12[e]	0.78 ± 0.10	0.83 ± 0.12	0.77 ± 0.11[e]	0.65 ± 0.09	0.768	0.432	0.417
Ferritin (ng/ml)	131.40 ± 55.03	123.71 ± 51.44	115.61 ± 50.99[b]	203.39 ± 50.56	173.11 ± 47.26[b]	191.73 ± 46.85	0.613	0.239	0.425

Values are expressed as adjusted means ± standard error of n=7 LCHF & n=8 HCLF. [b] $P < 0.05$, [bb] $P < 0.01$, denotes significantly different to baseline; [e] $P < 0.05$, denotes significantly different to endpoint. DBP, Diastolic blood pressure; HOMA IR, homeostatic model of insulin resistance; NEFA, non-esterified fatty acids; rQUICKI, revised Quantitative Insulin sensitivity Check Index; SBP, systolic blood pressure.

Supplement S3 – Dietary analysis results

Food diary analysis showed no significant ($P > 0.05$) differences in energy or macronutrient composition at baseline. As intended, the percentage of energy derived from fat and carbohydrate significantly ($P < 0.001$) increased from 34% to 61% and decreased from 42% to 10% respectively in the LC group. Whereas in the HC group, fat percentage decreased slightly from 36% to 33% and carbohydrate percentage remained unchanged at 41% which was significantly different from the LC group ($P < 0.001$) (Table 2). No significant differences were observed in energy intake between groups or throughout the intervention ($P > 0.05$). The percentage of total sugar derived from energy also significantly ($P < 0.001$) decreased in the LC group compared to the HC group. The increase in dietary fat in the LC group also led to a significant ($P < 0.01$) increase in energy derived from saturated (13% to 24%), monounsaturated (8% to 17%) and polyunsaturated (3% to 9%) fatty acids compared to the HC group. There was also a significant ($P = 0.04$) decrease in fibre intake in the LC group compared to the HC group which remained unchanged. The percentage energy of protein significantly ($P < 0.001$) increased within the LC group by 7% compared to the HC group which only increased by 2%.

Table S4. Changes in dietary composition with the LCHF and HCLF diets.

	LC			HC			(*P* value)	
**Nutrients**	**Week 0**	**Week 4**	**Week 8**	**Week 0**	**Week 4**	**Week 8**	**TIME**	**T X G**
Energy (kCal)	1974 ± 618	1970 ± 387	1728 ± 482	2263 ± 686	2165 ± 587	2304 ± 742	0.79	0.45
CHO (g)	218 ± 74	50 ± 18[bb]	45 ± 20[bb]	252 ± 189	249 ± 66	258 ± 113	<0.001	<0.001
CHO (%)	42 ± 9	10 ± 5[bbb]	10 ± 4[bb]	41 ± 8	43 ± 8	41 ± 6	<0.001	<0.001
Sugar (g)	70 ± 21	28 ± 11[bbb]	26 ± 13[bbb]	85 ± 45	95 ± 46	86 ± 49	<0.01	<0.001
Sugar (%)	15 ± 6	6 ± 2[bb]	6 ± 2[bb]	14 ± 4	17 ± 6	14 ± 4	<0.01	<0.001
Fibre (g)	22 ± 4	11 ± 4[bbb]	16 ± 10	25 ± 12	25 ± 8	27 ± 10	0.03	0.04
PRO (g)	88 ± 37	120 ± 26	106 ± 33	95 ± 20	95 ± 13	106 ± 23	0.09	0.08
PRO (%)	17 ± 3	25 ± 5[bb]	24 ± 4[bb]	17 ± 3	18 ± 3	19 ± 3	<0.001	<0.001
Fat (g)	75 ± 25	132 ± 49[b]	118 ± 39	89 ± 25	82 ± 30	85 ± 31	0.04	<0.01
Fat (%)	34 ± 4	58 ± 13[bb]	61 ± 6[bbb]	36 ± 7	33 ± 7	33 ± 7	<0.001	<0.001
SFA (g)	28 ± 9	56 ± 25	45 ± 18	28 ± 9	26 ± 11	28 ± 12	0.03	<0.01
SFA (%)	13 ± 3	24 ± 8[b]	23 ± 4[bb]	11 ± 3	10 ± 4	11 ± 3	<0.001	0.001
PUFA (g)	10 ± 4	20 ± 13	21 ± 10	12 ± 5	11 ± 4	10 ± 4	0.07	0.02
PUFA (%)	3 ± 1	7 ± 3[b]	9 ± 5[b]	4 ± 2	4 ± 2	4 ± 2	<0.01	<0.001
MUFA (g)	22 ± 13	41 ± 17	40 ± 15	24 ± 13	25 ± 13	21 ±9	0.03	0.02
MUFA (%)	8 ± 4	15 ± 8[b]	17 ± 7[bb]	8 ± 4	8 ± 5	7 ± 4	<0.01	<0.01

Values are expressed as means ± SD of n=8 LC & n=8 HC. [b] $P < 0.05$, [bb] $P < 0.01$, [bbb] $P < 0.001$, *denotes significantly different to baseline. CHO, Carbohydrates; MUFA, monounsaturated fatty acids, PUFA, polyunsaturated fatty acids; PRO, protein; SFA, Saturated fatty acids.*

Supplement S4 – Body Composition analysis

Body composition largely improved in the LC group but remained unchanged within the HC group over 8 weeks (Table 3). Body mass significantly (P < 0.001) decreased in the LC group and led to a significant (P < 0.001) decrease in BMI compared to the HC group which remained unchanged throughout. This decrease in body mass was largely attributed to a significant (P < 0.001) 3 kg reduction in fat mass in the LC group. A significant (P < 0.01) interaction in FFM was observed between groups, due to a small increase in FFM in the HC group and small decrease in the LC group. However, FFM % significantly (P = 0.02) increased in the LC group and remained (P = 0.53) unchanged in the HC group. At each time point FFM % was significantly (P < 0.05) higher in the HC group. Furthermore, skeletal muscle mass % did not significantly (P > 0.05) change with either diet and was only significantly (P = 0.02) different between groups at baseline. In the LC group, WC showed a tendency (P = 0.05) of decreasing, with post-hoc analysis showing a significant (P = 0.049) decrease at endpoint vs baseline only. No significant (P = 0.35) change was observed in the HC group and no significant (P > 0.05) difference was observed between groups at any time point. Similarly, In the LC group, VAT showed a tendency (P = 0.05) of decreasing, with post-hoc analysis showing a significant (P = 0.049) decrease at endpoint vs baseline only. No significant (P = 0.54) change was observed in the HC group and no significant (P > 0.05) difference was observed between groups at any time point.

Table S5. Changes in anthropometric variables following the LCHF and HCLF diets for 8 weeks.

	LCHF			HCLF				
**Measurement**	**Week 0**	**Week 4**	**Week 8**	**Week 0**	**Week 4**	**Week 8**	**TIME**	**T X G**
Mass (kg)	89.53 ± 14.85	86.51 ± 14.51[bb]	86.39 ± 14.25	72.48 ± 14.72	72.01 ± 14.88	72.27 ± 14.7	<0.001	<0.001
BMI (kg/m^2)	28.75 ± 2.15	27.76 ± 1.98[bb]	27.61 ± 1.26[b]	24.56 ± 3.83	24.40 ± 3.98	24.50 ± 3.98	<0.001	<0.001
FM (kg)	32.08 ± 4.28	29.80 ± 3.6[bb]	29.10 ± 3.41[bb]	19.89 ± 7.46	19.48 ± 7.89	19.60 ± 8.22	0.01	<0.001
FFM (kg)	57.39 ± 11.62	56.71 ± 11.87	57.28 ± 11.95	52.59 ± 11.63	52.52 ± 11.74	52.67 ± 11.54	0.008	0.003
			**Non-parametric testing**				**LCHF (Time)**	**HCLF (Time)**
FM (%)	34.40 ± 9.40	32.80 ± 7.50[bb]	33.30 ± 8.00[bb]	25.50 ± 2.50	24.90 ± 2.68	25.05 ± 3.88	0.021	0.530
FFM (%)	65.30 ± 9.00	67.20 ± 7.50	66.70 ± 8.00	74.50 ± 2.50	75.05 ± 2.67	74.95 ± 3.87	0.021	0.531
SkM (%)	33.00 ± 5.60	33.70 ± 6.20	33.30 ± 7.50	35.80 ± 4.20	36.25 ± 3.92	35.90 ± 4.37	0.258	0.768

Values are expressed as means ± SD of n=8 LCHF & n=8 HCLF. Non-parametric testing values are expressed as median ± IQR of n=7 LC & n=8 HC. [b] *$P < 0.05$,* [bb] *$P < 0.01$, denotes significantly different to baseline, *$P < 0.05$ denotes significantly different between groups at that timepoint.*

BMI, Body mass index; FM, Fat Mass; FFM, Fat-free mass; SkM, Skeletal Muscle.

Supplement S5 – Physical Activity analysis

There was no significant ($P > 0.05$) difference at baseline in total physical activity (TPA) (LCHF; 218 ± 44.1 min p/day vs HCLF; 216.15 ± 49 min p/day) or total moderate to vigorous intensity (MVPA) (LCHF; 214 ± 44.5 min p/day vs HCLF; 205.9 ± 48 min p/day). There was also no significant ($P > 0.05$) difference in TPA over 8 weeks (LCHF; 218 ± 44.1 to 270.81 ± 101.5 min p/day vs HCLF; 216.15 ± 49 to 222.7 ± 70.4 min p/day) between groups or MVPA (LCHF; 214 ± 44.5 to 266.5 ± 102.5 min p/day vs HCLF; 205.9 ± 48 to 209.2 ± 71.7 min p/day).

Table S6. The change in 78 metabolites following a LCHF and HCLF diet after 8 weeks

Metabolite	Fold change (%)	
	LCHF	HCLF
Hydroxybutyrylcarnitine	121.83	-5.03
Acetyl-L-carnitine*	47.95	1.03
Phytosphingosine-1-P	34.3	20.78
Phytosphingosine	30.6	-53.38
Hydroxyhexanoycarnitine	24.11	0.79
Galactosylceramide (d18:1/16:0)	21.99	-6.26
26-hydroxycholesterol 3-sulfate	15.44	-13.4
Ceramide (d18:1/24:0)	12.92	-11.66
cis-5-Tetradecenoylcarnitine	8.34	-14.88
3-hydroxyoctanoyl carnitine	8.15	-13.07
Galabiosylceramide (d18:1/16:0)	6.42	-11.82
Linoleyl carnitine	6.34	-9.07
Ceramide (d18:1/22:0)*	-1.65	-12.98
Dioleoylphosphatidylcholine	-5.03	-20.7
2-Oleoylglycerophosphocholine	-17.38	15.78
Glycerol trihexanoate	-19.28	15.45
Dodecanoylcarnitine	-20.64	-16.69
1-Linoleoylglycerophosphocholine	-39.62	-2.19
Linoleic acid	-49.42	-6.62
2,3-Diacetoxypropyl stearate	-60.99	28.91
20-Oxo-leukotriene E4	-99.99	ND
20-COOH-leukotriene E4	-100	ND
